# Apolipoprotein E expression is elevated by interleukin 1 and other interleukin 1-induced factors

**DOI:** 10.1186/1742-2094-8-175

**Published:** 2011-12-15

**Authors:** Ling Liu, Orwa Aboud, Richard A Jones, Robert E Mrak, W Sue T Griffin, Steven W Barger

**Affiliations:** 1 Department of Geriatrics, University of Arkansas for Medical Sciences, Little Rock AR 72205, USA; 2 Neurobiology and Developmental Sciences, University of Arkansas for Medical Sciences, Little Rock AR 72205, USA; 3 Geriatric Research Education and Clinical Center, Central Arkansas Veterans Healthcare System, Little Rock AR 72205, USA; 4 Department of Pathology, University of Toledo Health Science Campus, Toledo OH 43614, USA

**Keywords:** Alzheimer's disease (AD), amyloid beta (Aβ), apolipoprotein E (ApoE), beta amyloid precursor protein (βAPP), excitotoxicity, glutamate, interleukin-1 (IL-1β), neuroinflammation, neuronal stress, secreted amyloid precursor protein (sAPP)

## Abstract

**Background:**

We have previously outlined functional interactions, including feedback cycles, between several of the gene products implicated in the pathogenesis of Alzheimer's disease. A number of Alzheimer-related stressors induce neuronal expression of apolipoprotein E (ApoE), β-amyloid precursor protein (βAPP), and fragments of the latter such as amyloid β-peptide (Aβ) and secreted APP (sAPP). These stressors include interleukin-1 (IL-1)-mediated neuroinflammation and glutamate-mediated excitotoxicity. Such circumstances are especially powerful when they transpire in the context of an *APOE *ε4 allele.

**Methods:**

Semi-quantitative immunofluorescence imaging was used to analyze rat brains implanted with IL-1β slow-release pellets, sham pellets, or no pellets. Primary neuronal or NT2 cell cultures were treated with IL-1β, glutamate, Aβ, or sAPP; relative levels of ApoE mRNA and protein were measured by RT-PCR, qRT-PCR, and western immunoblot analysis. Cultures were also treated with inhibitors of multi-lineage kinases--in particular MAPK-p38 (SB203580), ERK (U0126), or JNK (SP600125)--prior to exposure of cultures to IL-1β, Aβ, sAPP, or glutamate.

**Results:**

Immunofluorescence of tissue sections from pellet-implanted rats showed that IL-1β induces expression of βAPP, IL-1α, and ApoE; the latter was confirmed by western blot analysis. These protein changes were mirrored by increases in their mRNAs, as well as in those encoding IL-1β, IL-1β-converting enzyme (ICE), and tumor necrosis factor (TNF). IL-1β also increased ApoE expression in neuronal cultures. It stimulated release of sAPP and glutamate in these cultures too, and both of these agents--as well as Aβ--stimulated ApoE expression themselves, suggesting that they may contribute to the effect of IL-1β on ApoE levels. Inhibitors of MAPK-p38, ERK, and JNK inhibited ApoE induction by all these agents except glutamate, which was sensitive only to inhibitors of ERK and JNK.

**Conclusion:**

Conditions of glial activation and hyperexcitation can elevate proinflammatory cytokines, ApoE, glutamate, βAPP, and its secreted fragments. Because each of these factors promotes glial activation and neuronal hyperexcitation, these relationships have the potential to sustain self-propagating neurodegenerative cycles that could culminate in a progressive neurodegenerative disorder such as Alzheimer's disease.

## Introduction

The pluripotent glial cytokine interleukin-1 (IL-1) and the CNS-abundant, lipid-cholesterol-carrying protein apolipoprotein E (ApoE) are key participants in the pathogenesis of Alzheimer's disease (AD). ApoE contributes both to learning and to recovery from neural injury [1], perhaps by enhancing synaptogenesis by influencing Reelin signaling [2,3]. In humans, single-nucleotide polymorphisms in the coding region of the ApoE gene (*APOE*) yield three alleles (ε2, ε3, ε4) that translate into three distinct protein sequences, ApoE2, ApoE3, and ApoE4. Inheritance of the particular isoform of ApoE encoded by the ε4 variant of the *APOE *gene confers significant risk for precocious development of AD [4,5]: those with two copies of the ε4 allele of *APOE *have a 50-90% chance of developing AD by the age of 85, and even one copy confers a three-fold increase in risk over individuals with no ε4 alleles [6]. Though ApoE is primarily expressed in astrocytes in the healthy brain, stressors can induce its expression in neurons [7,8].

Although not as strongly associated with AD risk as possession of ApoE4 sequences, specific polymorphisms in the genes encoding IL-1α and IL-1β are also associated with increased AD risk. Specifically, variations in the promoter region of *IL1A *and in the coding region of *IL1B *influence AD risk when homozygous in one gene or heterozygous in both [9-13]. Glial activation marked by excess production of both IL-1α and β is a constant feature in several conditions associated with increased risk for precocious development of AD: *i*) traumatic brain injury (TBI) [14], *ii*) systemic viral disease, e.g., AIDS [15]; *iii*) the neuronal hyperexcitability of epilepsy [16-19]; *iv*) chromosome 21 anomalies such as Down's syndrome [20]; and *v*) advancing age [21-23]. Each of these stressors is associated with precocious development of AD [18,24,25], especially in those who have inherited one or more ε4 alleles of *APOE *[1,26-29].

Excess production and secretion of IL-1β elevates neuronal expression of the precursors of each of the changes characteristic of AD. These neurodegeneration-related precursors include β-amyloid precursor protein (βAPP), which may lead in vivo to deposition of Aβ [30] and further induction of IL-1β [31]; ApoE, which is present in plaques [32] and necessary for the accumulation of Aβ deposits [33]; and hyperphosphorylated tau [5], the principal component of neurofibrillary tangles. IL-1 also induces α-synuclein [34], the Lewy body precursor.

Despite the potential for contributing to the production of Aβ, elevations of βAPP may participate in compensatory responses. βAPP is elevated in response to stressors beyond IL-1β, including excitotoxins and age itself, yet AD pathology is correlated with a deficiency in βAPP expression [35]. ApoE appears to mediate the compensatory induction of βAPP; blocking ApoE synthesis or its receptors inhibits the effect of glutamate on βAPP [35]. βAPP knockout mice show learning and memory deficits [36] and die prematurely [37]; secreted βAPP (sAPP) is generally neuroprotective [38]. Taken together, these findings suggest that possession of an ε4 allele or ApoE insufficiency compromises neurological parameters and exacerbates injury-induced deficits at least in part by limiting inductions of βAPP. ApoE, especially ApoE3, may also serve to keep inflammatory reactions in check [39-41]. A possible mechanism is suggested by the ability of ApoE to suppress the proinflammatory activity of sAPP [31].

In AD, activated microglia overexpressing IL-1 are present in diffuse Aβ deposits prior to the appearance of ApoE [32]. With normal aging, the brain shows increased microglial activation and expression of IL-1 [21], as well as neuronal expression of both ApoE and βAPP [35]. The ability of IL-1β to induce βAPP expression [30,42] raises the question of whether this is a direct mechanism or an indirect phenomenon resulting from ApoE induction, similar to the effect of glutamate [35]. In view of the relations between the AD-related stressors and the importance of ApoE in risk for development of AD, together with the neuropathological changes observed in AD patients, we tested the hypothesis that ApoE would be elevated in CNS neurons secondary to several AD-related stressors associated with excessive expression of IL-1. Specifically, rat primary cortical neurons and a neuropotent human cell line (NTera2) were assessed for ApoE expression after treatment with IL-1β, sAPP, glutamate, or Aβ. To delineate the roles of multi-lineage kinase (MLK) pathways in the induction of neuronal ApoE expression, we utilized inhibitors of p38-MAPK, ERK, and JNK pathways. To determine if such changes in ApoE expression might be observed in vivo, and the potential relationship of such changes to other proteins that are induced by IL-1, we measured the expression of ApoE, βAPP, and other neuroinflammatory proteins in rat brains exposed to excess IL-1β.

## Materials and methods

### Pellet Implantation

Pellets (1.5 mm in diameter, designed for controlled slow release of compounds over a 21-day period; Innovative Research of America, Sarasota, FL) impregnated with IL-1β (100 ng recombinant mouse IL-1β; Sigma Chemical Company, St. Louis, MO) and 'control' pellets (with bovine serum albumin) were implanted 2.8 mm caudal to bregma, 4.5 mm right of the midline, and 2.5 mm below the pial surface. Twenty-one male Sprague-Dawley rats, weighing 264 ± 6 g, were randomly assigned to three groups. Eight rats received implants of 21-day timed-release IL-1β-containing pellets, seven rats received sham (vehicle) pellets, and six rats served as unoperated controls. Twenty-one days after implantation, cortices from left hemispheres were collected for protein and mRNA isolation. For histological study, brain tissues were fixed in 10% formalin, embedded in paraffin, sectioned at 7 μm, and prepared for immunohistochemical analysis. All animal studies were conducted in accordance with a protocol reviewed and approved by the Institutional Animal Care and Use Committee of the Central Arkansas Veterans Healthcare System.

### Reagents

Rat recombinant mature IL-1β (IL-1β holoprotein cleavage product) was purchased from Sigma (St. Louis MO), secreted APP (sAPP) was purified from a recombinant expression system as described previously [42], and L-glutamate was from Sigma (St. Louis MO). Aβ_1-42_, from US Peptide Inc. (Rancho Cucamonga CA), was dissolved in DMSO and then incubated at 4°C overnight prior to use. Rabbit anti-mouse IL-1β antibody was from Chemicon (Temecula CA); goat anti-human apolipoprotein E was from Calbiochem (Sunnyvale CA). Inhibitors of the p38-MAPK (SB203580), ERK (U0126), and JNK (SP600125) pathways were from Calbiochem. Medium, serum, and B27 supplement for cell cultures were from Invitrogen/Life Technologies (Grand Island NY). The antibodies used were rabbit anti-human IL-1α (Peprotech 4:1000), goat anti-human APP (ADI 1:50), goat anti-Human APO E (Invitrogen 1:50), diluted in antibody diluent (Dako, Carpiteria CA).

### Immunofluorescence

For immunofluorescent analysis of brain tissues, paraffin blocks were sectioned at 7 μm and placed on pre-cleaned microscope slides (Fischer). Then, sections were deparaffinized in xylene, rehydrated in graduated ethanol solutions to deionized water. For IL-1α immunoreactions, sections were placed in boiling sodium citrate buffer (0.01 M, pH 6.0) for 20 minutes. Sections for βAPP and ApoE were placed in trypsin solution for 10 minutes at 37°C, all sections were blocked using protein block (Dako). For each antibody, sections were incubated overnight at room temperature. The secondary antibodies, Alexa Fluor donkey anti-goat and donkey anti-rabbit were diluted in antibody diluent (Dako) and sections were incubated for 60 minutes. The sections were then washed in three changes 5 minutes each of distilled H_2_O and then coverslipped with prolong Gold with DAPI (Invitrogen).

### Image Analysis

Similar to our previous study [35], a quantitative approach was used to examine mean intensities of immunoreactions. Three representative images per slide (40× magnification) from IL-1-pellet, sham, and unoperated rat brains were obtained at identical exposure settings, using a Nikon Eclipse E600 microscope equipped with a Coolsnap monochrome camera. Each of the three images in each tissue section spanned a total area of 37241.5 μm^2^. These images were from hippocampal CA1 and two cortical regions, one at the midline and another at the superior aspects of the temporal cortex and were acquired and analyzed using NIS-Elements BR3 software (Nikon.com). All cells of a type were captured, and images were thresholded. Data obtained from cells in each of the three regions were averaged, thus providing a single value for each image, and this value was used for statistical analysis. Data were analyzed by ANOVA to assess difference among groups. A statistical value of p ≤ 0.05 was defined as being significant.

### Cell Cultures

Primary neuronal cultures were derived from cerebral cortex of fetal Spraque-Dawley rats (embryonic day 18), as previously described [43]. Experiments using primary neuronal cell cultures were performed after 10-14 days in culture. Highly purified cultures of rat microglia and astrocytes were generated from the cortical tissue of neonatal (0-3 days) Sprague-Dawley rats, as described previously [43,44]. The NTera2 human cell line (Stratagene, La Jolla, CA) was maintained in Dulbecco's modified Eagle medium (DMEM; Invitrogen/Life Technologies, Grand Island, NY) supplemented to 10% with fetal bovine serum (FBS). For specific experiments, SB203580 (5 μM), U0126 (5 μM), or SP600125 (5 μM) was applied to cultures one hour before application of a stimulus. Glutamate released in the culture medium was assayed with a kit that utilizes a glutamate dehydrogenase-coupled color reaction (Roche Diagnostics, Indianapolis IN).

### Reverse Transcription (RT) Reaction and Polymerase Chain Reaction (PCR) Amplification

Total RNA was extracted from cultured cells using TriReagent™ RNA (Molecular Research Center Inc., Cincinnati OH) according to the manufacturer's instructions. Gel-based RT-PCR was performed as described previously [45]. Briefly, RT reactions were performed simultaneously using reagents from a single master mix, and PCR was performed using reagents from Clontech (Palo Alto CA). Aliquots of the product were resolved on agarose gels, ethidium bromide staining was captured by digital camera, and pixel intensities were quantified with Scion Image 4.0.3.2. Conditions were established to ensure that maximal cycle number fell within the linear phase of amplification. Real-time (quantitative) RT-PCR was performed as described previously [35]. RT utilized random hexamers for priming, and PCR was performed with the Power SYBR-Green PCR Master Mix in an ABI 7900 HT Fast Real-time PCR System (Applied Biosystems, Foster City, CA). Signals were interpolated within standard curve reactions performed for each primer set, and the result for ApoE was expressed as a fraction of the 18S signal for each sample. All primer sequences, annealing temperatures, and number of cycles are provided in Table 1.

**Table 1:** RT-PCR primers

Gene	Sequences	Annealing temp. (°C)	**Cycle no**.
Gel-based PCR, rat			

ApoE	F: TGT TGG TCC CAT TGC TGA CAG GAT	60	25
			
	R: TGG TGT TTA CCT CGT TGC GGT ACT		

βAPP	F: CCC TGA CGC AGT CGA CAA GT	61	25
			
	R: TGT CAT AAC CTG GGA CCG GAT		

GAPDH	F: CAA CGG ATT TGG CCG TAT TG	61	25
			
	R: TGG GGG TAG GAA CAC GGA A		

ICE	F: ACA AAG AAG GTG GCG CAT TTC	61	28
			
	R: CCT TGT TTC TCT CCA CGG C		

IL-1α	F: GAG TCA ACT CAT TGG CGC TTG	60	27
			
	R: GGG CTG ATT GAA ACT TAG CCG		

IL-1β	F: TGA CTC GTG GGA TGA TGA CG	61	27
			
	R: CTG GAG ACT GCC CAT TCT CG		

TNF	F: GCA CAG AAA GCA TGA TCC GAG	64	28
			
	R: CCT GGT ATG AAG TGG CAA ATC G		

Gel-based PCR, human			

ApoE	F: TTC TGT GGG CTG CGT TGC TG	60	25
			
	R: TAC ACT GCC AGG CGC TTC TG		

GAPDH	F: AGG TCG GAG TCA ACG GAT TTG	61	25
			
	R: TGG CAG GTT TTT CTA GAC GGC		

Real-time PCR (rat)			

ApoE	F: CTG GTT CGA GCC GCT AGT G	60	N.A.
			
	R: CCT GTA TCT TCT CCA TTA GGT TTG C		

18S	F: TTC GAA CGT CTG CCC TAT CAA	60	N.A.
	R: ATG GTA GGC ACG GCG ACT A		

### Western Immunoblot Assay

Cellular fractions were prepared by application of a lysis buffer (50 mM Tris-HCl, pH 7.5, 150 mM NaCl, 1% Nonidet P40, 0.5% sodium deoxycholate and 0.1% SDS) to the cultures after a wash with cold PBS. Tissue samples were prepared by homogenization in RIPA buffer (Cell Bioscience) as described previously [35,42]. Lysates were quantified using a Micro BCA assay reagent kit (Pierce, Rockford IL) as described previously [43]. Aliquots (100 μg each) were resolved by SDS-PAGE, subjected to electrophoresis at 70 V for 20 minutes and 90 V for 1.5 h, and transferred to nitrocellulose membranes. After transfer, each blot was stained with Ponceau S to ensure even loading of protein across lanes. Blots were then blocked in I-Block Buffer (Applied Biosystem Inc., Bedford, MA) for 45 minutes, then incubated overnight at 4°C with goat anti-human ApoE (1:2000) primary antibody, incubated for 1 h at room temperature with alkaline phosphatase-conjugated secondary antibody, and developed using the Western-Light™ Chemiluminescent Detection System (Applied Biosystem Inc) and exposure to x-ray film. Digital images were captured and analyzed using NIH Image software, version 1.60.

### Statistical Analysis

Comparisons between two conditions were made via unpaired *t*-test, and experiments with a greater number of variables were subjected to ANOVA with Fisher's *post hoc *test. Differences were considered significant at *p*-values ≤0.05.

## Results

### Chronic IL-1β increases the expression of ApoE, βAPP, and neuroinflammatory factors in rat brain

**Figure 1:**
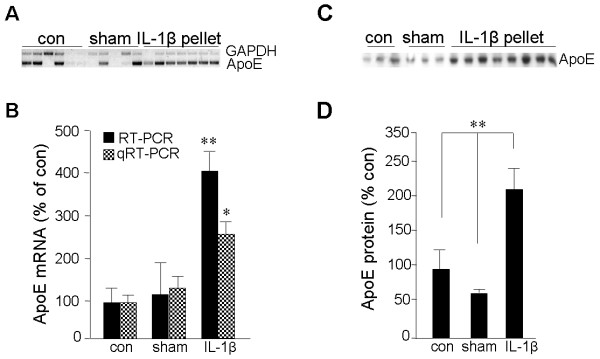
**IL-1β induces ApoE expression *in vivo***. Proteins were extracted from the left cerebral cortex of unoperated rats (**con**), of rats implanted in the right cerebrum with a pellet containing vehicle alone (**sham**), and of rats implanted in the right cerebrum with a slow-release pellet containing 100 ng IL-1β (**IL-1β pellet**). **A: **RNA was extracted from the left cerebral cortex of con (n = 6), sham (n = 5), and IL-1β pellet (n = 7) rats and subjected to gel-based RT-PCR for ApoE and GAPDH. **B: **Quantification of the ApoE mRNA (relative to that for GAPDH) by densitometry of the RT-PCR image or by quantitative (real-time) RT-PCR (**qRT-PCR**); values reflect the percent of control as mean ± SEM. **C: **Proteins were extracted from con (n = 3), sham (n = 3), and IL-1β pellet (n = 8) rats and subjected to western immunoblot analysis for ApoE. **D: **Quantification of autoradiography; values reflect percent of control as mean ± SEM. *p≤0.05; **p < 0.01 versus control.

Rats were implanted with either slow-release (21-day) IL-1β-impregnated pellets or vehicle-impregnated sham pellets. Cerebral cortices from these rats, as well as unoperated control rats, were processed for protein or mRNA tissue level analyses or were fixed and processed for immunofluorescent image analyses. Rat brains implanted with IL-1β-containing pellets had markedly elevated steady-state levels of ApoE mRNA (Figure 1A,B) and of ApoE protein (Figure 1C,D) compared to those in rats implanted with sham pellets or to unoperated controls (*p *< 0.01).

**Figure 2:**
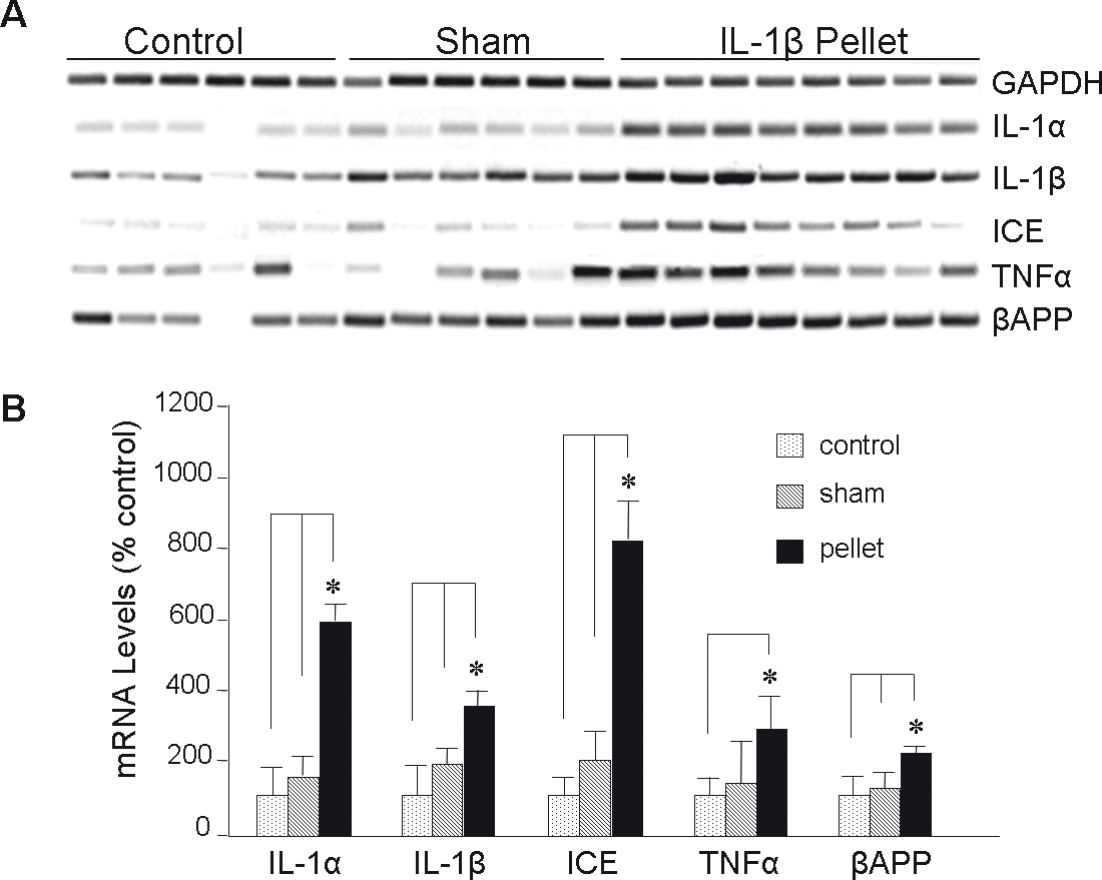
**IL-1β induces increases in mRNAs encoding ApoE, βAPP, and the proinflammatory factors IL-1β, ICE, IL-1α, and TNF**. RNA was extracted from the left cerebral cortex of unoperated rats (**Control**), rats implanted in the right cerebrum with a pellet containing vehicle alone (**Sham**), and rats implanted in the right cerebrum with a slow-release pellet containing 100 ng IL-1β (**IL-1β Pellet**); **A: **semi-quantitative RT-PCR was performed on the RNA. **B: **Results quantified by densitometry showed significant differences in mRNA levels from brains with IL-1β pellets compared to those with sham pellets, or from those serving as controls; *p*≤0.05 for each gene product.

Neuroinflammatory conditions and models thereof often exhibit chain reactions of multiple effectors working sequentially, in parallel, or in feedback loops fomenting a persistent and progressive situation. In this vein, the ability of IL-1β to elevate the levels of IL-α prompted an examination of gene-expression indices of neuroinflammation in this chronic IL-1β delivery paradigm. The increase in IL-1α immunofluorescence noted above was found to be reflected at the mRNA level (Figure 2). Chronic IL-1β also elevated mRNA levels of endogenous IL-1β, as well as its cleavage enzyme ICE (Figure 2). Along with these changes in IL-1-related molecules, the mRNA for the proinflammatory cytokine TNF was elevated. These proinflammatory changes were accompanied by induction of βAPP mRNA (Figure 2), consistent with the immunofluorescence results and prior studies of IL-1/βAPP interactions.

**Figure 3:**
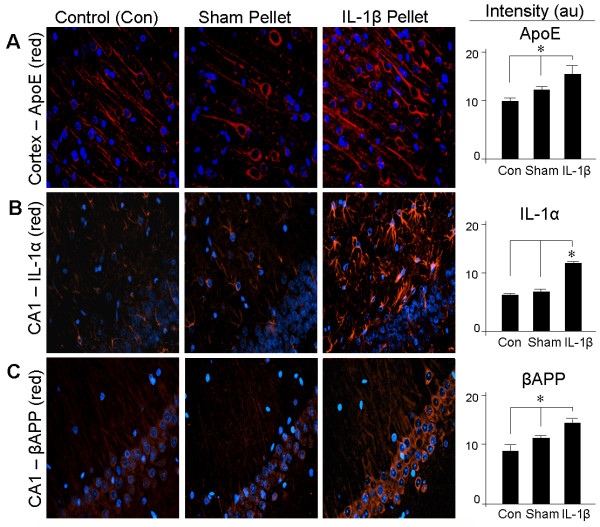
**IL-1β induces expression of IL-1α, ApoE, and βAPP**. Immunofluorescence was performed on tissue sections from the neocortex or hippocampus (CA1) of unoperated rats (n = 4) (**Control**), from rats implanted with a pellet containing vehicle alone (n = 6) (**Sham**), and from rats implanted with a slow-release pellet containing 100 ng IL-1β (n = 6) (**IL-1β Pellet**). Primary antibodies were **A: **ApoE, **B: **IL-1α, or **C: **βAPP; the red immunofluorescence signal is countered by a blue DAPI stain of cell nuclei. Immunofluorescence was quantified as described in Materials and Methods, and is expressed as mean ± SEM. All treatment conditions were significantly different from each other except with regard to IL-1α, where only the comparison of IL-1β pellet to the other two treatments was significant (*p ≤0.05).

The induction of ApoE in the cortex by IL-1β pellets was also detectable by immunofluorescence (Figure 3A), which demonstrated neuronal localization. IL-1β pellets also elevated expression of IL-1α in the CA1 of hippocampus (Figure 3B). This IL-1α induction was localized principally to cells with astrocytic morphology. Pyramidal neurons of the CA1 overexpressed βAPP in response to the chronic delivery of IL-1β (Figure 3C).

### Tissue culture studies reveal potential for indirect impacts of IL-1β on ApoE

**Figure 4:**
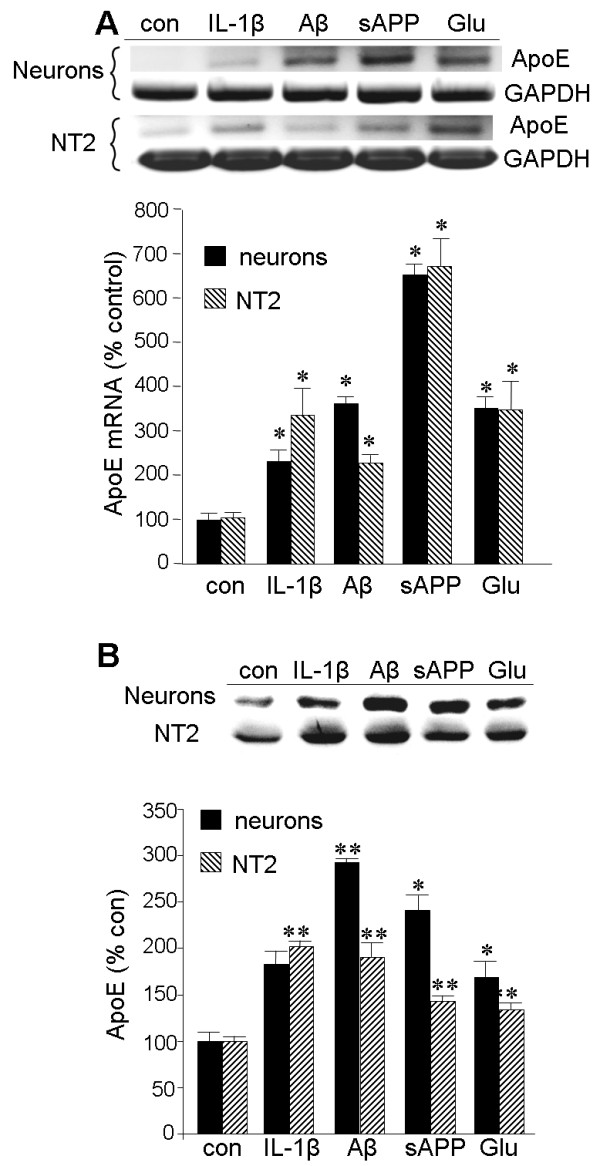
**Elevation of ApoE expression in neuronal cells by stimuli relevant to Alzheimer's disease**. Primary cortical neurons or NT2 cells were treated with IL-1β (30 ng/ml), Aβ_1-42 _(10 μM), sAPP (30 nM), or Glu (50 μM) for 20 h. **A: **RNA was extracted from the cultures for RT-PCR of ApoE and GAPDH as illustrated. The results were quantified by densitometry, and values represent the mean ± SEM for the ApoE amplimer relative to that for GAPDH. **B: **Cell lysates from the cultures were equilibrated for total protein levels and analyzed for ApoE content by western blot, also quantified by densitometry (*p < 0.05, **p < 0.01).

To examine the impact of IL-1β on ApoE expression in greater temporal and mechanistic detail, we utilized two types of neuronal cell culture: primary cultures of rat cortical neurons and the human NTera2 (NT2) cell line. We previously demonstrated that glutamate elevates βAPP expression via a mechanism that requires the biological activity of ApoE [35]. Moreover, IL-1β has been shown to influence the processing of βAPP [46]. Therefore, we tested whether ApoE expression was responsive to these agents and another derivative of βAPP: Aβ_1-42_. In both culture types, expression of ApoE mRNA was elevated approximately two-fold by exposure to IL-1β, Aβ_1-42_, or glutamate for 20 h; the induction by sAPP exceeded six-fold (Figure 4A). All of these agents were found to elevate ApoE protein levels as well (Figure 4B).

**Figure 5:**
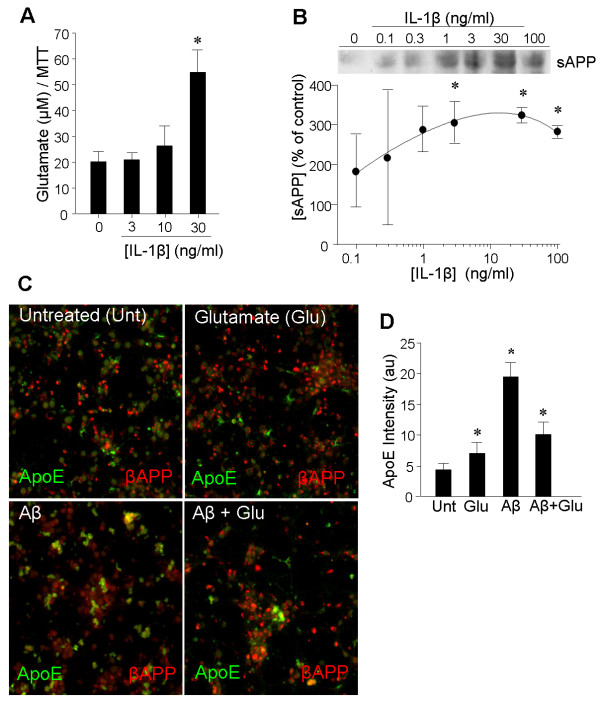
**Potential for indirect effects of IL-1β**. **A: **Primary cortical neurons were treated for 20 h with the indicated concentrations of IL-1β, and glutamate levels were assessed in the medium (*p < 0.05). **B: **Primary cortical neurons were treated for 20 h with the indicated concentrations of IL-1β, and sAPP levels were assessed in concentrated aliquots of the culture medium by western blot; *p < 0.05. **C: **Primary cortical neurons were treated for 20 h with glutamate (50 μM) and Aβ_1-42 _(10 μM) alone, or with a combination of the two; immunofluorescence was performed for ApoE (green) and βAPP (red). **D: **The bar graph shows integrated fluorescence intensity quantified for the ApoE signal in the cultures shown in panel C; n = 3 cultures for each treatment (*p < 0.05 compared to the untreated sample).

The ability of glutamate and βAPP fragments to impact ApoE was given additional relevance by demonstration of impacts of IL-1β on these agents. Levels of glutamate released into neuronal culture medium was elevated by IL-1β (Figure 5A). Likewise, IL-1β elevated the levels of sAPP in the culture medium of primary neurons in a dose-dependent fashion (Figure 5B). Glutamate induction of ApoE in primary neurons was confirmed by immunofluorescence, which also documented a larger induction by Aβ_1-42 _(Figure 5C,D). Intriguingly, coapplication of glutamate in combination with Aβ_1-42 _reduced the induction to one on par with that of glutamate alone (Figure 5D).

### Regulation of ApoE expression by IL-1β, Aβ, sAPP, and glutamate is via multi-lineage kinase pathways

**Figure 6:**
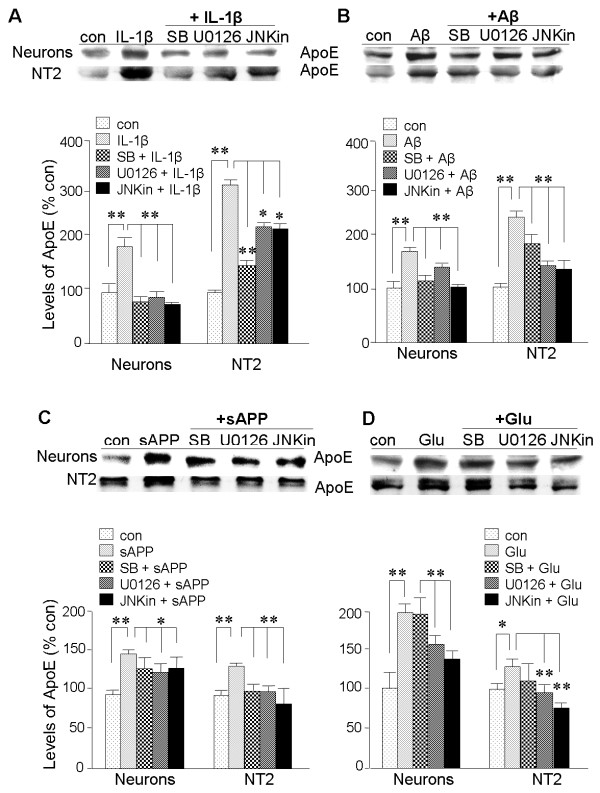
**Roles for p38-MAPK, ERK, and JNK in elevation of ApoE expression by IL-1β, Aβ_1-42_, sAPP, and glutamate**. Cultures of primary cortical neurons or NT2 cells were treated for 20 h with **A: **IL-1β (30 ng/ml), **B: **Aβ_1-42 _(10 μM), **C: **sAPP (30 nM), **D: **or glutamate (50 μM). In parallel cultures, these agents were preceded by 5 μM of an inhibitor of p38-MAPK (SB), the ERK pathway (U0126), or JNK (JNKin). Lysates were examined by western blot analysis of full-length ApoE, and autoradiographs were quantified by densitometry (bar graphs). Values represent mean ± SEM (n = 4); *p < 0.05, **p < 0.01.

Each of the IL-1β-induced entities, sAPP and glutamate, as well as Aβ, were shown to elevate ApoE expression in both primary neurons and NT2 cells (Figure 5). To begin investigating the mechanisms involved in the induction of such ApoE expression, we focused on multi-lineage kinases (MLKs) previously shown to regulate cytokine-induced AD-related proteins [47]. Primary neurons and NT2 cells were incubated with inhibitors of three principle MLK pathways, viz., the MEK/ERK (U0126), MAPK-p38 (SB203580), and JNK (SP600125) pathways. Constitutive expression of ApoE in both primary neurons and NT2 cells was unaffected by treatment with these inhibitors (data not shown). However, each of these MLK inhibitors suppressed induction of ApoE by IL-1β (Figure 6A), Aβ_1-42 _(Figure 6B), and sAPP (Figure 6C) in both types of culture. Induction of ApoE by glutamate in both NT2 and primary neurons was not inhibited by SB203580, a MAPK-p38 inhibitor. Thus, regulation of ApoE expression by MLK pathways appears to be somewhat selective and dependent on the effector of its induction; in the case of glutamate, ERK and JNK activity is involved but not MAPK-p38 (Figure 6D).

## Discussion

The neuroinflammagenic potential of IL-1β is shown here through its induction of synthesis of itself and other proinflammatory cytokines including TNF, IL-1α, IL-1β, as well as the latter's maturation enzyme ICE. The additional impact of IL-1β on neuronal ApoE production shown here suggests that in neurological conditions where the expression of proinflammatory cytokines is elevated, the expression of IL-1-driven AD-related proteins such as ApoE would be elevated as well. Multiple MLKs--ERK, p38-MAPK, and JNK--were shown to be involved in elevated expression of ApoE in neurons exposed to IL-1β, Aβ, or sAPP. The increased expression of ApoE induced by glutamate was mediated by ERK and JNK, but not by MAPK-p38. Together, these findings have several implications for AD pathogenesis, particularly with respect to conditions in which neuroinflammation is prominent, especially those influenced by *APOE *genotype.

The actions of IL-1 and the other agents tested here--sAPP, Aβ, and glutamate--create the possibility for complex loops of influence analogous to the vicious circle of neuroinflammatory events we have termed the Cytokine Cycle [48]. Glutamate can elevate neuronal expression of βAPP and its conversion to sAPP [42]. βAPP is elevated in dystrophic neurites in and around plaques [49]; its breakdown into both sAPP and Aβ can result in induction of IL-1β in microglia [31,50]. In addition to inducing IL-1β expression and release, sAPP and Aβ also stimulate microglia to release biologically relevant levels of glutamate and its cooperative excitatory amino acid D-serine [44,51,52]. Thus, future studies should address the potential for each of these agents to act indirectly through the elaboration of a key agent or agents that can directly stimulate ApoE expression.

Key to interpretation of our findings--and, indeed, to the role of *APOE *genotype in AD--is determining whether elevation of ApoE levels would be beneficial or harmful. Possession of the ε4 allele is associated with enhanced deposition of Aβ [53], consistent with *in vitro *studies wherein ApoE was shown to enhance Aβ fibrillogenesis [54]. In this regard, ApoE4 has been shown to be more effective than ApoE3, fostering speculation that replacement of the ε3 allele by ε4 merely enhances an activity already present in ApoE3. This has been described as a toxic gain of function, implying that overabundance of any ApoE--even ApoE2 or ApoE3--would also create a gain in this function and thus be detrimental. Moreover, transient increases in cellular ApoE occur in response to injuries that promote AD, e.g., traumatic brain injury [55] and stroke [56]. ApoE4 is generally reported to be present at higher steady-state levels than ApoE3 in CSF or brain parenchyma [57-61], though some studies have reported lower levels of total ApoE in ε4-positive individuals [62,63].

In contrast to these connections to pathology, ApoE provides neuroprotection in many paradigms, and ApoE deficiency has proved detrimental in several respects [64]. Therefore, inductions of ApoE by the stimuli we tested may represent a compensatory response, meaning that the distinction between ApoE3 and ApoE4 represents loss of a beneficial function. ApoE has anti-inflammatory effects, and even its interaction with Aβ can attenuate glial activation by the latter [65]. However, ApoE3 is more effective than ApoE4 as an anti-inflammatory agent [31,65,66], so this putative compensatory response may be inadequate in ε4-positive individuals and thus allow more extensive propagation of the Cytokine Cycle. Such an allele-specific compensatory response may also extend to direct neuroprotective activity. We previously reported that ApoE3 induces βAPP expression but ApoE4 does not [35], confirming the findings of Ezra *et al. *[67]. In this regard, elevations of ApoE by the process of neuroinflammation, or other stressors, would reflect a requisite role for the lipoprotein in enhancing the beneficial roles of βAPP and/or other acute-phase response proteins. Thus, it would be the inability of ApoE4 to participate in this chain of salutary events that makes it detrimental. We have previously shown that the increase in ApoE brain levels that occurs with aging continues to occur in AD, with a large fraction being deposited in plaques [35]. This increase in ApoE levels is distinguishable from changes in βAPP, which rises with age but declines markedly in AD [35]. This disease-associated severance of the coordinate regulation of ApoE and βAPP further strengthens the correlation of brain health with the coregulation of these two proteins; to wit, with ApoE expression itself, provided that the ApoE is not ApoE4.

Multi-lineage kinase pathways may be invoked in the regulation of ApoE expression, and can themselves be invoked by ApoE [68,69], suggesting a feedback loop between MLK pathways and ApoE expression in neurons. Our findings of involvement of multiple MLKs--ERK, p38-MAPK, and JNK--in expression of ApoE in neurons exposed to IL-1β, Aβ, or sAPP, together with previous reports of ERK pathway invocation of ApoE expression and vice versa, are consistent with the existence of a complex feedback system that may be important in acute-phase responses to neuronal injury as well as potential exacerbation of neurodegenerative events. Our finding that glutamate regulates ApoE expression via ERK and JNK, but not by p38-MAPK pathways may be indicative of a correlation between glutamatergic induction of ApoE and neuronal survival. Excitotoxic effects of glutamate are largely dependent upon activation of extrasynaptic NMDA receptors, p38-MAPK, and the inhibition of ERK signaling; synaptic receptors, on the other hand, appear to activate ERK and promote survival [70-72].

In conclusion, the induction of neuronal ApoE by either neuroinflammatory or excitotoxic agents or neurotoxins, acting through MLK pathways suggests that alterations in these signaling pathways, together with other neuropathological entities in AD brain, may have consequences for ApoE expression. Differences in this expression may be critical, considering the role of *APOE *genotype in AD risk. The response of ApoE to IL-1β we show here in rodent brain suggests that elevation of IL-1 leads to the increases in ApoE that we and others have observed in the AD brain. This may have added significance with regard to the self-propagating nature of IL-1-driven cascades, especially when such cascades are instigated in the context of an ε4 allele of *APOE*. While induction of ApoE2 or ApoE3 may be anti-inflammatory or neuroprotective, and thereby act as a self-limiting influence on IL-1-driven cascades, ApoE4 may fail to participate and leave the brain vulnerable to prolonged activation of a maladaptive cycle.

## Competing interests

S.W.B. receives royalty payments from Sigma-Aldrich Corp., which manufactures some of the reagents utilized in this study. The authors have no other conflicts to declare.

## Authors' contributions

LL performed the cell culture experiments and contributed to writing the first draft of the manuscript. OA performed the immunofluorescence and assisted with western blots and writing. RAJ performed the experiments with rat brain tissue. REM contributed to interpretation of the results and to writing. WSTG contributed to the design of the study, interpretation of the results, and writing. SWB made essential contributions to the design of the study and interpretation of the results and completed the final draft of the manuscript. All authors read and approved the final manuscript.
